# Data in support of intermolecular interactions at early stage of protein/detergent particle association induced by salt/polyethylene glycol mixtures

**DOI:** 10.1016/j.dib.2016.04.003

**Published:** 2016-04-08

**Authors:** Takayuki Odahara, Koji Odahara

**Affiliations:** a National Institute of Advanced Industrial Science and Technology (AIST), Tsukuba Central-6, 1-1 Higashi, Tsukuba, Ibaraki 305-8566, Japan; b Fukuoka Agriculture and Forestry Research Center, Chikusion, Fukuoka 818-8549, Japan

**Keywords:** Integral membrane protein, Phase separation, Polyethylene glycol, Salt, Stability

## Abstract

The data provide information in support of the research article, “Intermolecular interactions at early stage of protein/detergent particle association induced by salt/polyethylene glycol mixtures” [1]. The data regarding variation of absorption spectra is used as an indicator of the duration of *Rp. viridis* PRU and RC, *Rb. sphaeroides* RC and LH2, and *Rb. capsulatus* LH2 in the native state in the presence of NaCl/polyethylene glycol (PEG) mixture. The data about minimum concentrations of salt and PEG whose aqueous phases are mutually separated presents information on additional influence of Tris buffer and N-octyl-β-d-glucoside on the salt–PEG phase separation.

**Specifications table**

**Table:** 

Subject area	*Biophysics*
More specific subject area	*Association of protein/detergent particles by salt/PEG mixtures*
Type of data	*Figure*
How data was acquired	*Absorption spectroscopy for protein stability, and observation with eyes for salt/PEG phase separation*
Data format	*Scaled intensity data for absorption spectra, and raw data for salt/PEG phase separation*
Experimental factors	*Wild-type photosynthetic bacteria were obtained from ATCC. Chemicals employed were high-grade ones; polyethylene glycol 4000 for gas chromatography was purchased from MERCK, NaCl and Tris(hydroxymethyl)aminomethane for biochemical assay from Wako, N-octyl-*β*-d-glucoside and N-dodecyl-*β*-d*-*maltoside from DOJINDO, and N,N-dimethyldodecylamine N-oxide from SIGMA*
Experimental features	*Absorption spectra were measured at various time points after NaCl/PEG mixture addition. Minimum concentrations for immiscible aqueous phases of salt and PEG were determined in the presence of 25 mM Tris buffer and 8 mg/mL OG.*
Data source location	*Tsukuba, Japan*
Data accessibility	*Data are available in this article.*

## Value of the data

• Protein stability is a significant factor for determination of measurement time points after precipitant addition in the study of association of proteins in the native states.
• Protein stability will also provide basic information for the study of denaturation process of proteins caused by salt/PEG mixtures.
• Influence of buffer and detergent on salt–PEG phase separation is basic information to avoid the undesired influence on the association of integral membrane proteins.

## 1 Data

In this data article, data are shared regarding protein stability and salt–polyethylene glycol (PEG) phase separation. The former is absorption spectra of *Rp. viridis* PRU [2], [3] and RC, *Rb. sphaeroides* RC [4], [5] and LH2, and *Rb. capsulatus* LH2 measured at different time points after addition of NaCl/PEG mixture. The latter is shown as minimum concentrations of salts and PEG that form immiscible aqueous phases [6] in the presence of 25 mM Tris buffer and 8 mg/mL N-octyl-β-d-glucoside.

## 2 Experimental design, materials and methods

### 2.1 Stability of integral membrane proteins in the presence of NaCl/polyethylene glycol mixture

**Fig. 1:**
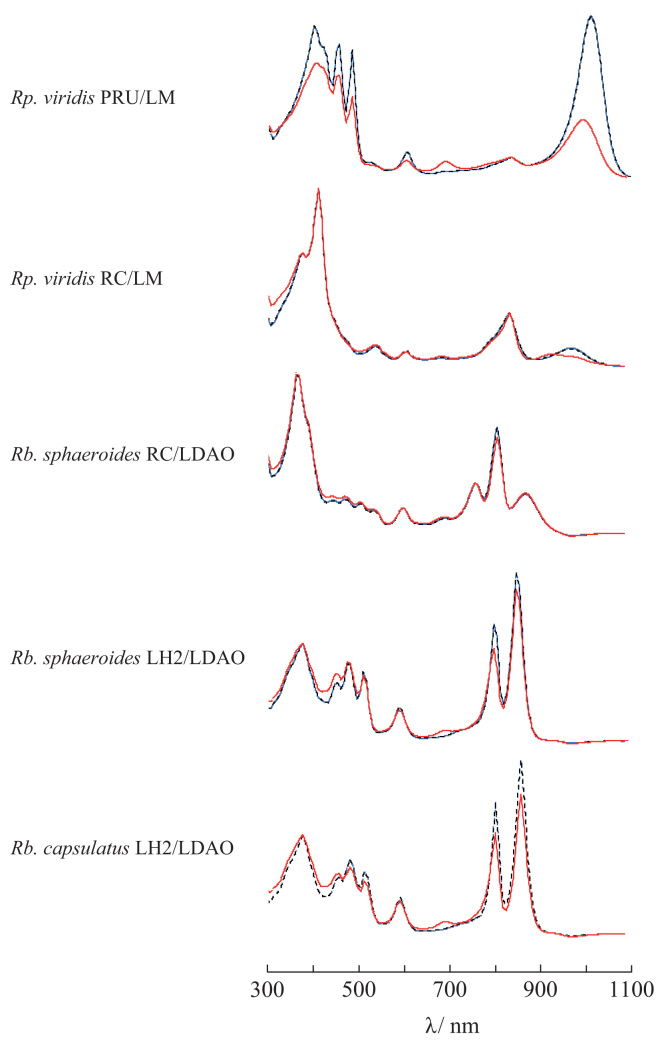
Absorption spectra of integral membrane proteins from photosynthetic bacteria stored at 20 °C in darkness in the presence of NaCl/PEG mixture. The examined proteins were *Rp. viridis* PRU and RC, *Rb. sphaeroides* RC and LH2, and *Rb. capsulatus* LH2. The spectra of each protein were measured for the purified sample (blue line) and the supernatants within 1 h (black dotted line) and in 14–35 days (red line) after the mixture addition. The longer duration was 14, 15, 21 35 and 28 days for *Rp. viridis* PRU and RC, *Rb. sphaeroides* RC and LH2, and *Rb. capsulatus* LH2, respectively. The spectra were measured for protein solutions diluted by detergent-containing buffer solution (25 mM Tris–HCl and 300 mM NaCl; pH 8.0) of which the maximum absorbance was below 2. The spectra exhibited were scaled so as to fit their base lines to one another. The PEG concentration in the original supernatant was 62.5 mg/mL for *Rp. viridis* PRU/LM, 70 mg/mL for *Rp. viridis* RC/LM, 160 mg/mL for *Rb. sphaeroides* RC/LDAO, 150 mg/mL for *Rb. sphaeroides* LH2/LDAO, and 150 mg/mL for *Rb. capsulatus* LH2/LDAO. The concentration of LM and LDAO was 1 mg/mL.

Fig. 1 shows representatives of the spectra measured at various time points after the addition of NaCl/PEG mixture. At one hour or shorter time points after the mixture addition, no variations in the spectra were observed for all the proteins. After several to 30 days, however, four proteins excluding *Rb. sphaeroides* RC exhibited variation in their absorption spectra that reflected variation of the intramolecular cofactors and the peptides supporting them. With *Rp. viridis* PRU, the absorption band with a maximum at 1006 nm, arising from bacteriochlorophyll in the LH1 subunits, decreased and a new peak appeared at 687 nm. In the spectra of *Rp. viridis* RC, the absorption band with a maximum at 830 nm, arising from special pair of bacteriochlorophyll, disappeared. With *Rb. sphaeroides* LH2 and *Rb. capsulatus* LH2, the two absorption peaks at 800 nm and 850 nm decreased and a small peak appeared at 690 nm.

### 2.2 Influence of Tris buffer and N-octyl-β-d-glucoside on phase separation of salt and polyethylene glycol

**Fig. 2:**
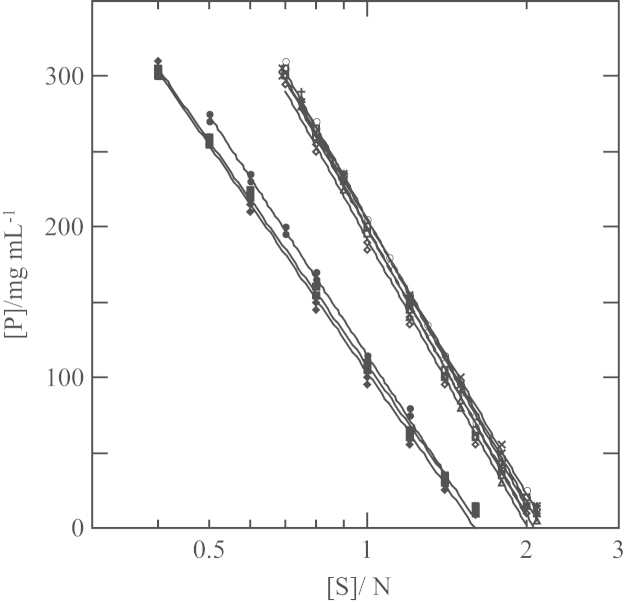
Minimum PEG concentrations at various salt concentrations for the formation of immiscible salt- and PEG-aqueous phases in the presence of 25 mM Tris buffer and 8 mg/mL OG at room temperature (21–24 ^°^C). The salts examined were di-potassium tartrate, di–potassium hydrogen phosphate, and tri–potassium citrate. The solution ingredients were potassium tartrate in water (○), 25 mM Tris–HCl (□) and 25 mM Tris–HCl and 8 mg/mL OG (◇), K_2_HPO_4_ in water (•), 25 mM Tris–HCl (■) and 25 mM Tris–HCl and 8 mg/mL OG (♦), and potassium citrate in water (✕), 25 mM Tris–HCl (+) and 25 mM Tris–HCl and 8 mg/mL OG (Δ).

Minimum PEG concentrations for phase separation at various salt concentrations were determined in the presence of 25 mM Tris buffer and 8 mg/mL OG, as follows. A concentrated salt solution, a 625 mg/mL PEG solution, a 400 mM Tris–HCl solution (pH 8.0), a 200 mg/mL OG solution and pure water were put in small glass tubes at different ratios, and the mixture was shaken vigorously on a vortex mixer. Formation of mutually immiscible phases was judged by observing with eyes whether the resultant mixtures were turbid. The result is shown in Fig. 2. Two symbols at each salt concentration in the figure represent the highest PEG concentration where clear (single-phase) solutions were formed and the lowest PEG concentration where turbid solutions (containing two immiscible phases) were formed. The true minimum PEG concentrations for the phase separation should be of values between the two PEG concentrations. With each of the three salts, the minimum PEG concentration [P] varied linearly against the logarithm of the salt concentration [S]. Hence, the [P]–[S] line for each salt was calculated by least-square fitting of the relationship, [P]=*A*_ps_−*B*_ps_ log [S], to the two PEG concentrations at various salt concentrations, where *A*_ps_ and B_ps_ were constants. The addition of Tris–HCl and OG exhibited an effect to lower the minimum PEG concentrations by 20 mg/mL at maximum. This effect was approximately similar to or less than the effect of the three salts at 25 mN.
